# *In Silico* Investigation of Cytochrome P450 2C9 in relation to Aging Using Traditional Chinese Medicine

**DOI:** 10.1155/2014/404505

**Published:** 2014-05-08

**Authors:** Tzu-Chieh Hung, Chia-Chen Kuo, Calvin Yu-Chian Chen

**Affiliations:** ^1^Department of Biomedical Informatics, Asia University, Taichung 41354, Taiwan; ^2^School of Medicine, College of Medicine, China Medical University, Taichung 40402, Taiwan

## Abstract

Cytochrome P450 2C9 (CYP2C9) metabolizes dehydroepiandrosterone-sulfate (DHEA-S), but in elderly people the amount of DHEA-S remaining after CYP2C9 metabolization may be insufficient for optimal health. A prediction model, molecular docking, and molecular dynamics were used to screen the Traditional Chinese Medicine (TCM) database to determine molecular compounds that may inhibit CYP2C9. The candidate compounds apocynoside(I), 4-methoxymagndialdehyde, and prunasin have higher Dock Scores, and prediction bioactivity than warfarin (the control drug). The interaction between 4-methoxymagndialdehyde and CYP2C9 is more intense than with other TCM compounds, but the simulation is longer. In these compounds, apocynoside(I) and prunasin have a greater number of pathways for their flexible structure, but these structures create weak interactions. These candidate compounds, which are known to have antioxidation and hypolipidemic functions that have an indirect effect on the aging process, can be extracted from traditional Chinese medicines. Thus, these candidate compounds may become CYP2C9 inhibitors and play an important role in providing optimal health in the elderly.

## 1. Introduction

Cytochrome P450 2C9 (CYP2C9) is an enzyme of the Cytochrome P450 superfamily of monooxygenases [1]. The four subfamilies of CYP are involved in different drug-metabolism processes, and both CYP2C9 and cytochrome P450 2C19 (CYP2C19) have major clinical functions [2]. CYP2C9 can metabolize a large range of therapeutic drugs, such as those involved with blood sugar regulation, anticoagulants, and the weak acid or base types of drugs [3–5]. CYP2C9 is involved in an NADPH-dependent electron transport pathway in liver microsomes [6].

Dehydroepiandrosterone (DHEA) is a human antistress 19-carbon steroid hormone [7], secreted mainly by the adrenal glands [8]. Most DHEA is secreted in the form of dehydroepiandrosterone-sulfate (DHEA-S) into the circulatory system [9] and converted to either androgens or estrogens [10–12]. DHEA thus has many functions, such as sex-hormone production, stress regulation, neural activity affection, neurotransmitter metabolism [13], and the prevention of brain aging [14]. DHEA secretions become maximal in the mid-twenties and then steadily decline over the next decades to around half the youthful value over the age of 45. This phenomenon will induce a loss of disease-resistance. Reports in the literature claim that CYP2C9 metabolizes DHEA-S, thus creating a reduced level of circulating DHEA in elderly people and, consequentially, causing aging and lowered disease resistance [15, 16]. Therefore it would seem that the inhibition of CYP2C9 can increase the level of DHEA in the circulatory system and reduce geriatric problems [7, 8, 17, 18].

Computer-Aided Drug Design (CADD) is an* in silico* simulation technique that has become popular in the pharmaceutical industry due to its low cost and speed of obtaining results. In this investigation CCAD was accomplished by using a molecular simulation of drug design and incorporating structure-based and ligand-based investigations.

The main aspects of simulation are molecular docking, bioactivity prediction, and molecular dynamics. The screening and selection of compounds was based on the above technique and the analysis of protein-ligand interactions [19–21].

Traditional Chinese medicine (TCM) plays an important role in health care in Asia. The TCM Database@Taiwan (http://tcm.cmu.edu.tw/) is the world's largest TCM database [22] and contains approximately 61,000 TCM compounds, as well as including information on the structure, bioactivity, and molecular data. The drug design application of the TCM Database@Taiwan has been confirmed by the phosphodiesterase-5 block [23], epidermal growth factor receptor (EGFR) inhibition [24], HER 2 receptor inhibition [25], inflammation inhibition [26], stroke prevention [27, 28] and against virus [29–31]. The TCM database is using cloud-computing web server for application, now [32, 33].

In this study the possible compounds were screened against CYP2C9 from the TCM Database@Taiwan. After molecular docking, the selected compounds are detected based on their bioactivity calculated by the support vector machine (SVM) and multiple linear regression (MLR) prediction models. Molecular dynamics (MD) was used to investigate the variation of protein-ligand interactions. This work can make a contribution to the assessment of the effects of CYP2C9 inhibition.

## 2. Materials and Methods

### 2.1. Data Collection

The molecular simulations were performed by Accelrys Discovery Studio 2.5 (DS 2.5). A total of 61,000 TCM compounds were downloaded from the TCM database (http://tcm.cmu.edu.tw/). The sequence of CYP2C9 was generated from Uniprot (http://www.uniprot.org/, CYP2C9_HUMAN, P11712) and the 3D crystal structure (PDB: 1OG5) was downloaded from the Protein Data Bank (http://www.rcsb.org/pdb/home/home.do). Warfarin is a generally available drug for the treatment of cardiovascular disease [34–36] and this drug was defined as the control drug [37, 38].

### 2.2. Disorder Protein Detection

Disordered proteins play an important role in drug design; thus, drug efficacy is based on protein structure and the ligand-interacting docking site [39, 40]. The disordered region could be predicted through the protein sequence of CYP2C9 submitted to the Database of Protein Disorder (DisProt, http://www.disprot.org/).

The docking site of CYP2C9 was based on the references for warfarin interactions with Arg97, Phe100, Ala103, Arg108, Phe110, Val113, Phe114, Asn204, Ile205, Leu208, Asn217, Val237, Met240, Val292, Asp293, Gly296, Ala297, Thr301, Leu366, Pro367, and Phe476 in CYP2C9 [37, 38].

A comparison of the disordered region and the docking site could help in the evaluation of the protein-ligand interaction.

### 2.3. Molecular Docking

The molecular simulations were performed using LigandFit, which is a receptor-rigid docking algorithm program in Accelrys Discovery Studio 2.5 (DS 2.5) [41]. In this program, warfarin and TCM compounds dock to CYP2C9 in the force field of CHARMm [42].

### 2.4. Ligand-Based Prediction

Bioactivity prediction was assessed by the MLR and SVM models. The pIC_50_ of 19 CYP2C9 inhibitors was set as the template to assist with model assessment (Table S1, see the Supplementary Material available online at http://dx.doi.org/10.1155/2014/404505) [43]. Before creating the prediction model, the descriptors of the ligand were evaluated through the Genetic Approximation (GA) algorithm of the Calculate Molecular Properties module in Accelrys Discovery Studio 2.5 (Table S2) [44].

The MLR was established by the five descriptors and the Matlab Statistics Toolbox was used to select the ligand based on activity [45] and was detected by the Leave One Out Validation [23]. The equation is as follows:

(1) y=b0+b1∗X1+b2∗X2+⋯+b5∗X5,

where *Y* is the pIC_50_ prediction result, *X* is descriptor, and *b* is a coefficient in the equation.

The SVM model used the same ligand template and descriptors. The descriptors should be normalized to transform the range from −1 to 1. Fivefold Cross Validation was used to screen the best training model [46]. The equation is as follows:

(2) Yi=(αi,xi)+b,(x,α)=∑αiki(x)+b,

where *k*_*i*_(*x*) represents a set of kernel transformations, *i* = 1,…, *m*, *α*_*i*_ is a vector of the linear function, and *b* is a coefficient.

The SVM regression model is determined by the *ε*-insensitive loss function:

(3) Lε(y,f(x,α))=0, if  |y−f(x,α)|≤ε|y−f(x,α)|−ε, otherwise.

After prediction, we selected the top 1–3 ligands based on data and employed these compounds as candidates for MD simulation.

### 2.5. Molecular Dynamics Simulation

In the Standard Dynamics Cascade and Dynamics (Production) package of DS 2.5 [19, 47], minimization was set to the steepest descent and the conjugate gradient set to a maximum of 500 steps. The heating time was 50–310 K, the equilibration time was 200 ps, and the total production time was 20 ns, with using NVT and the constant temperature dynamics used the Berendsen weak coupling method. The temperature coupling decay time was 0.4 ps with the Berendsen thermal coupling method. The target temperature was 310 K. After MD simulation [48], hydrogen bonds, the distance of the hydrogen bond, root mean square deviations (RMSD) of the complex, RMSD of the ligand, and total energy of the complex were analyzed by the analysis trajectory of DS 2.5. Finally, to depict the pathway of the ligand's movement into the docking site and run the protein after interaction, we calculated the aperture of the protein and the molecular structure of the ligand [49].

## 3. Results and Discussion

### 3.1. The Detection of Disorder Protein

**Figure 1:**
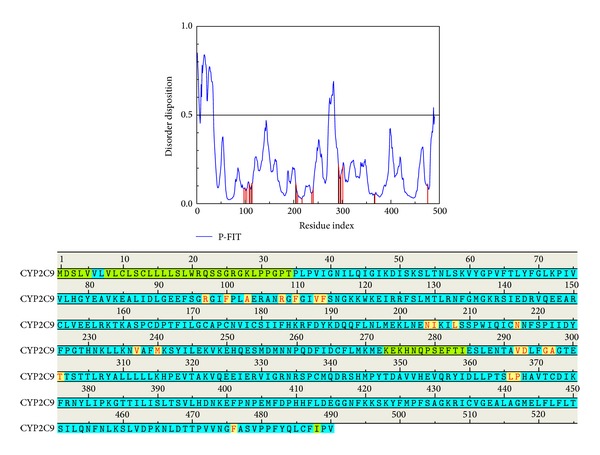
The disorder prediction and binding site detection. The blue curve indicates the disorder disposition of each amino acid and the red lines are the residues of the important amino acids. The amino acid sequence describes the information for the disorder regions. The green regions show the predicted disordered regions and the yellow regions, with the amino acids noted in red, contain important amino acids.

The disordered protein is intrinsically an unstructured protein and while the docking site consists of a disordered region the complex will only stabilize with difficultly. The disordered regions of CYP2C9 are defined as those regions with a disposition greater than 0.5 (Figure 1). This result indicates that the important amino acids do not consist of disordered regions; thus, the ligand docks to the appropriate selected site and our results have a weaker effect compared to the disordered protein. Consequently, the compounds selected were based on docking that could have an influence on CYP2C9.

### 3.2. Bioactivity Prediction by MLR and SVM Models

The GA algorithm can determine the optimal relationship between pIC50 and molecular descriptors. The top ten selected models have five molecular descriptors. For example, the correlation coefficient (*r* ^2^) of the top model is 0.9581, confirming that this model is credible (Table S2). The equation for the model is shown below:

(4) GFATempModel1=9.3969+4.6637∗ALogP−1.5025∗ESSumdssc−4.0939∗LogD+0.0089932∗JursDPSA1−0.27175∗Jurs_WPSA_3,

in which *A*Log*P* measures the hydrophobic surname of the molecule. The ES_Sum_dssc is the calculation of the E-state sum for atom type dssc. Log*D* is the water partition coefficient calculated by taking into account the ionization states of the molecule, and the default pH is 7.4. The Jurs_DPSA1 is the difference in charged partial surface areas. The Jurs_WPSA_3 is the surface-weighted charged partial surface area.

**Figure 2:**
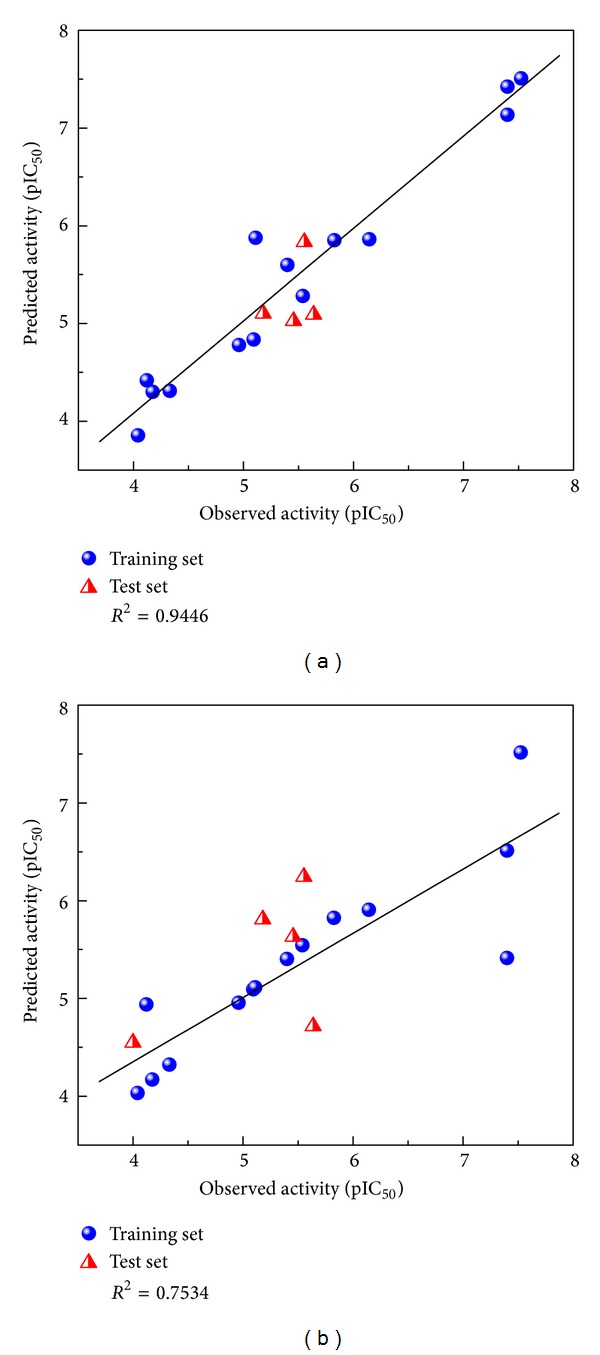
Relation of observed activity (pIC_50_) and predict activity (pIC_50_). (a) MLR. (b) SVM.

The results show the MLR and SVM models, with correlation coefficients of 0.9446 and 0.7534, respectively (Figure 2). These models are used to predict the bioactivity of the control and the TCM compounds. Finally, the top models were selected as candidates based on the SVM prediction (under the condition that docking score and SVM were better than in the control) (Table 1).

### 3.3. Molecular Docking

**Figure 3:**
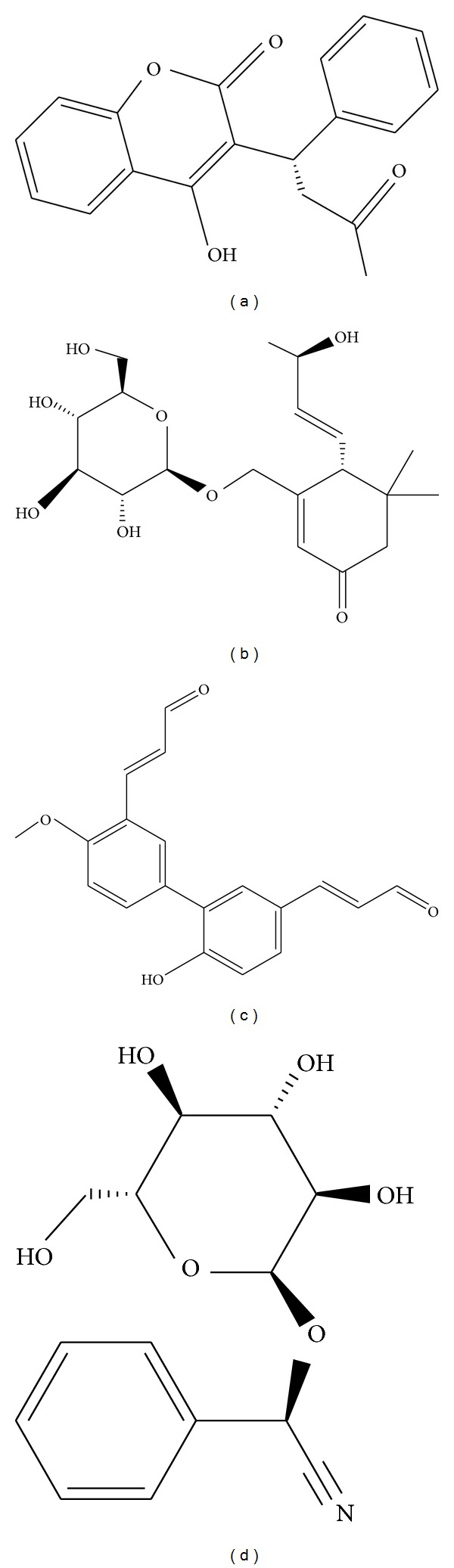
The structure of the control and the top three selected TCM compounds: (a) warfarin, (b) apocynoside(I), (c) 4-methoxymagndialdehyde, and (d) prunasin.

The top three compounds selected by the threshold, which requires that both the docking score and the bioactivity predicted from SVM are higher than the control, are apocynoside(I), 4-methoxymagndialdehyde, and prunasin. The 2D structure of the top three candidates, and warfarin, are presented in Figure 3. The top TCM candidate is apocynoside(I), extracted from* Apocynum venetum* L. while 4-methoxymagndialdehyde (top 2) is extracted from* Magnolia officinalis* Rehd. et Wils., and prunasin (top 3) is extracted* Citrus aurantium* L. These compounds have been confirmed for antioxidant and anti-inflammatory actions [50–54], which are important aspects of antiaging treatment. It could be suggested that the selected compounds, having these functions, may have an influence on CYP2C9.

**Figure 4:**
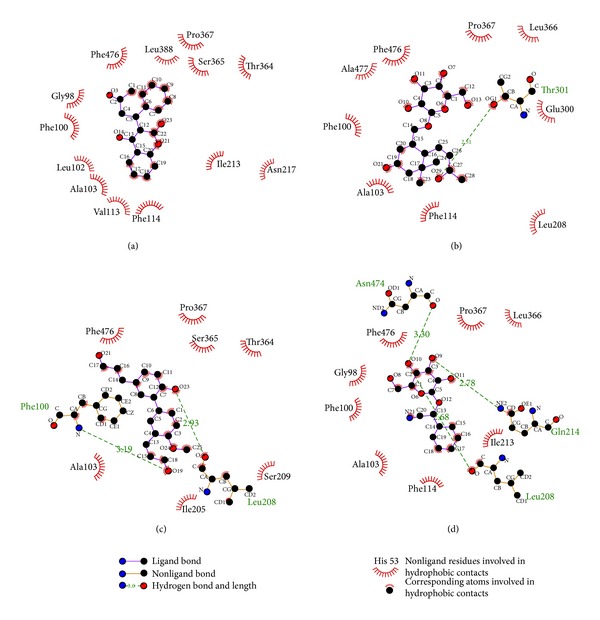
The hydrophobic interaction with the ligands in docking poses: (a) warfarin, (b) apocynoside(I), (c) 4-methoxymagndialdehyde, and (d) prunasin.

The hydrophobic interaction analysis was calculated by Ligplot v.2.2.25 to interpret docking poses (Figure 4). The results shown in Figure 4 indicate that the top three compounds have hydrogen bonds (H-bonds) but only warfarin has a hydrophobic interaction. Each docking score for the top three compounds is higher than the control because of their stronger interaction in the docking site.

**Figure 5:**
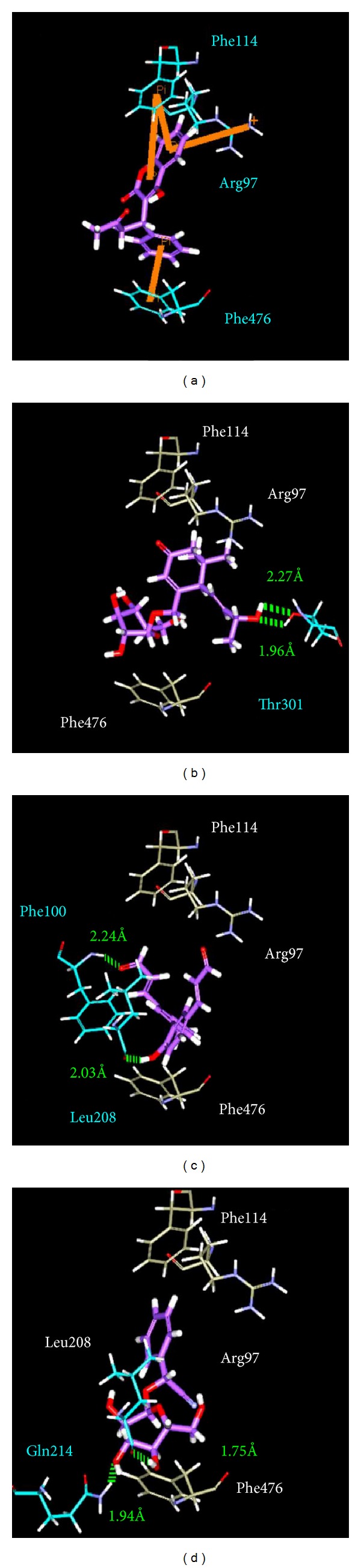
The H bonding between protein and ligand interaction in docking poses: (a) warfarin, (b) apocynoside(I), (c) 4-methoxymagndialdehyde, and (d) prunasin. The ligand is shown in purple, orange represents pi-pi interactions, cyan shows the control pi-pi interactions bonding amino acids, white is the nonbonding amino acids of the ligand, and green is H-bonds.

An interesting result in the docking poses presented by DS 2.5 (Figure 5) is that warfarin has a pi bond interaction with some of the twenty-one amino acids. It is well known that the pi bond is stronger than the H-bond but the docking score is not presented. In the observation of the distance between ligand and protein, warfarin is greater than the TCM compounds and warfarin has a fewer amino acids around the ligand than the TCM compounds. Based on the above reasons, we suggest that the docking score will be calculated according to the ligand's interaction with the protein's docking site.

### 3.4. Molecular Dynamics Simulation

**Figure 6:**
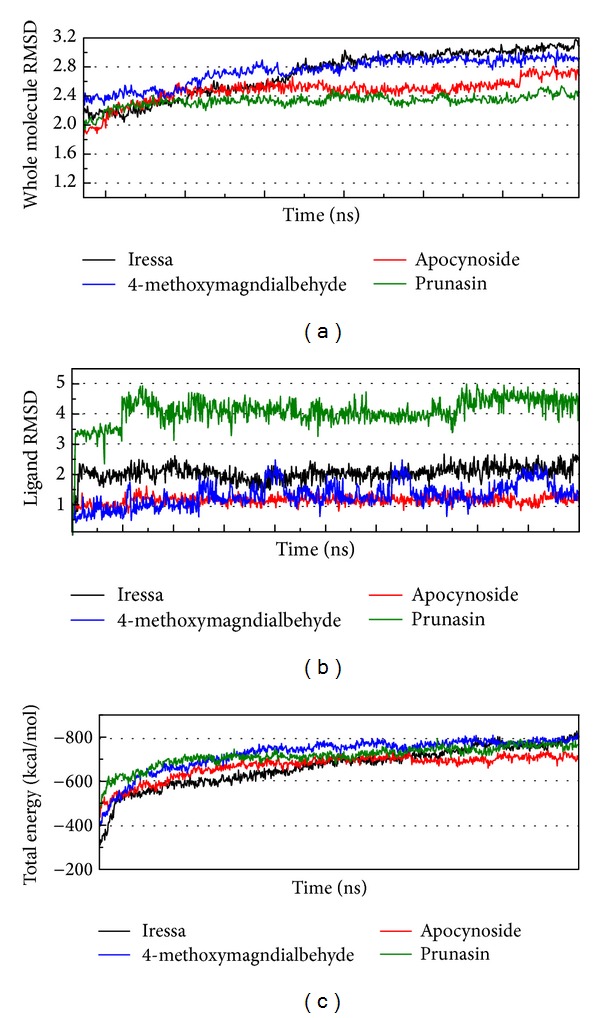
MD trajectories depicting changes during 20 ns simulation: (a) plot of complex RMSD, (b) plot of ligand RMSD, and (c) plot of complex total energy verses MD simulation time.

After an MD of 10 ns, the RMSD of the ligand, the RMSD of the complex, and the total energy are recorded (Figure 6). The RMSD of the complex and the total energy tend to lessen. This result indicates that the protein-ligand interaction will become balanced (4-methoxymagndialdehyde as ligand) at 20 ns of simulation. Interestingly, in Figure 6, the ligand RMSD of prunasin is the highest, but the complex RMSD and total energy are the lowest. To solve this problem, we will use the analysis of the protein-ligand interaction and the protein structure variation.

**Figure 7:**
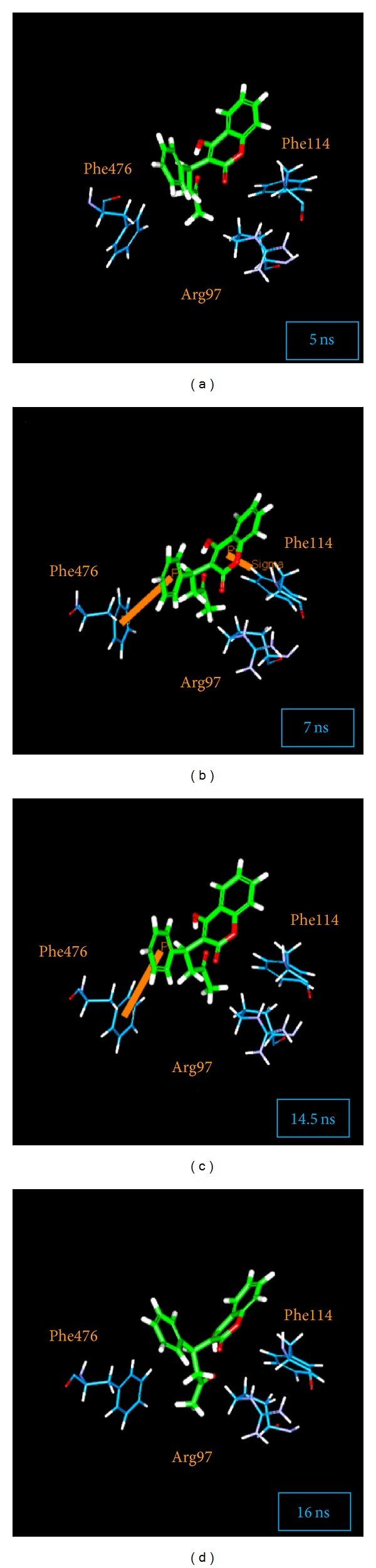
The pi-pi interaction during the warfarin MD: (a) disappears at 5 ns, (b) present at 7 ns, (c) present at 14.5 ns, and (d) disappears at 16 ns.

We captured the simulation PDB file based on the significant variation of RMSD and total energy (Figure 7). We noted that the pi bond in warfarin and in the CYP2C9 complex was not stable after making the comparisons between 5 ns, 7 ns, 14.5 ns, and 16 ns. The fact that the docking score for Warfarin was lower than TCM complex can be confirmed based on this result.

**Figure 8:**
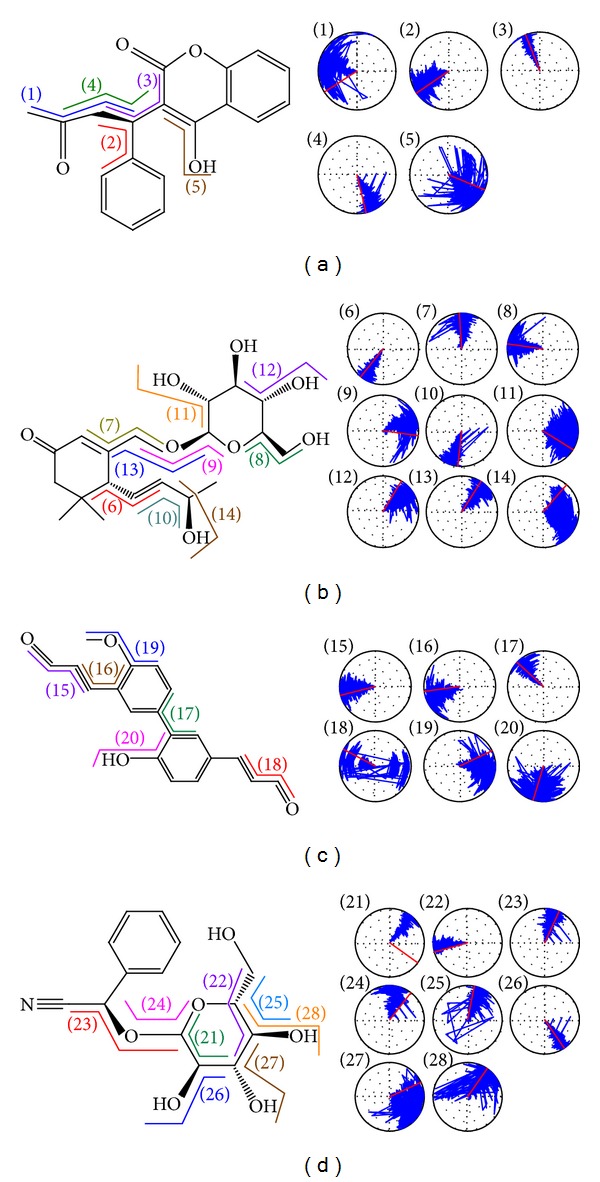
The torsion angle variation during 20 ns MD simulation. Each torsion angle is specified numerically and corresponds to the radar chart with the identical number: (a) warfarin, (b) apocynoside(I), (c) 4-methoxymagndialdehyde, and (d) prunasin.

Torsion helps to describe the variation of a protein structure (Figure 8). In this result, we can determine that the site involved with H-bonds will have an intense variation, such as torsions 1 and 5 in warfarin, torsions 11 and 14 in apocynoside(I), torsions 19 and 20 in 4-methoxymagndialdehyde, and torsions 27 and 28 in prunasin. This result shows that the H-bond in the warfarin complex is less than the observations for the frequency and amplitude in torsion.

**Figure 9:**
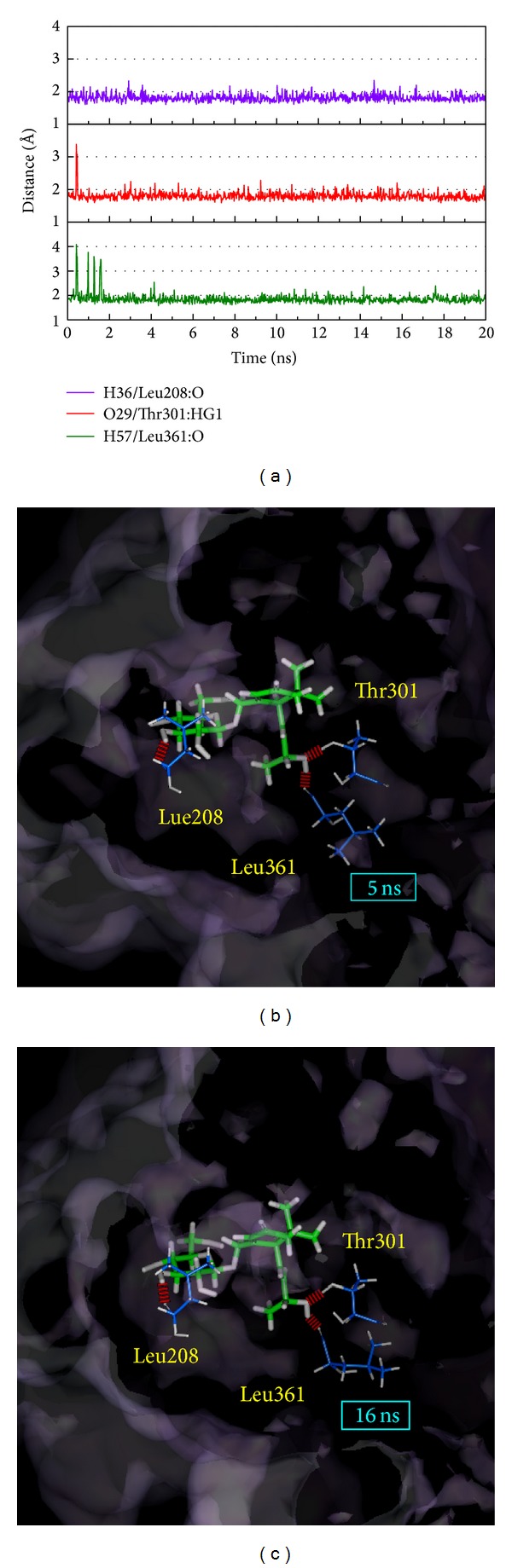
The variation of H-bond distance of the apocynoside (I) and CYP2C9 interaction in MD. (a) The top three (Leu208, The301, and Leu361) H-bond occupancies and the distance variations of the poses in (b) 5 ns and in (c) 16 ns.

**Figure 10:**
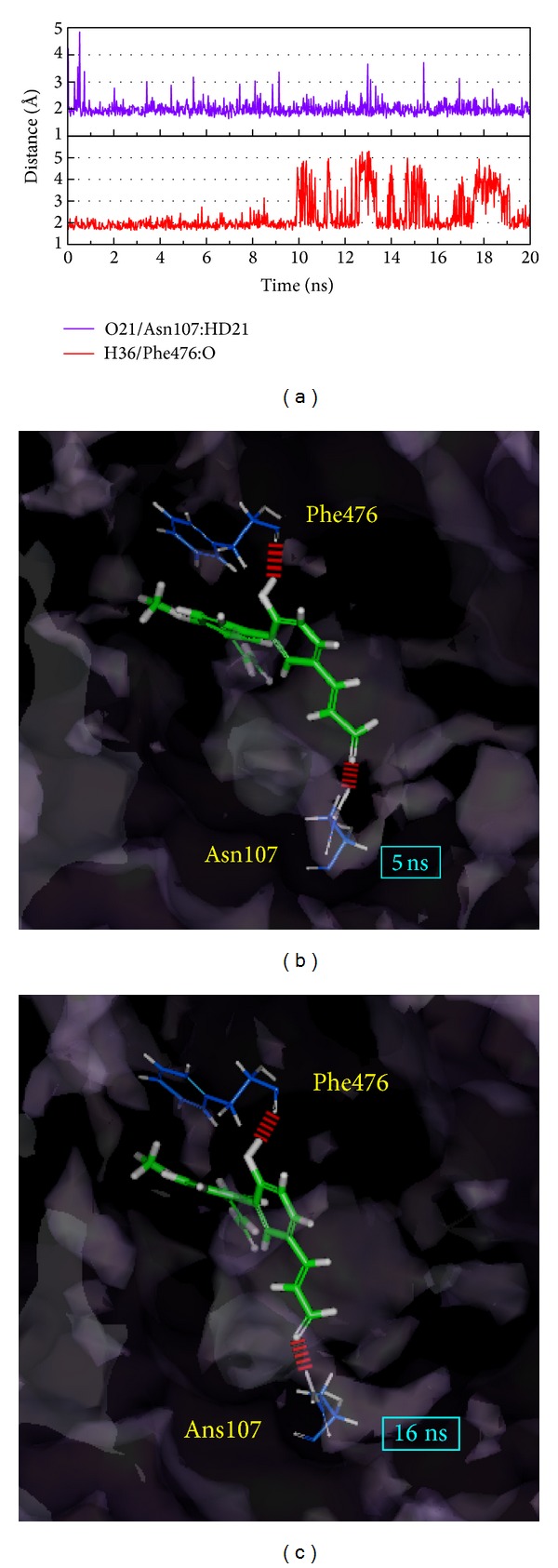
The variation in H-bond distance for the 4-methoxymagndialdehydein and CYP2C9 interactions in MD. (a) The top two (Asn107 and Phe476) H-bond occupancies and their distance variations in (b) 5 ns and in (c) 16 ns.

**Figure 11:**
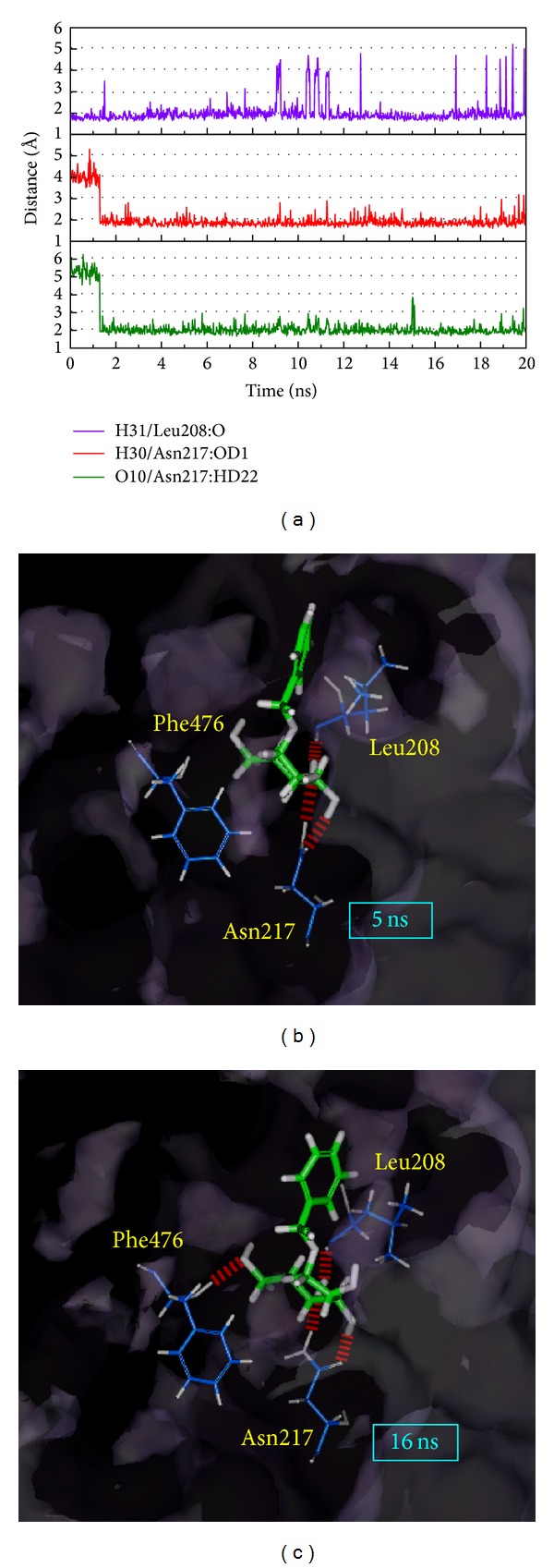
The variation in H-bond distance of the Prunasin and CYP2C9 interactions in MD. (a) The top two (Leu208 and Asn217) H-bond occupancies and their distance variations in (b) 5 ns and in (c) 16 ns.

We selected the occupancy of H-bonds to be greater than 50% in each TCM complex and we inspected the H-bond distance (Figures 9–11). In these results, the 5 ns and 16 ns data were captured on behalf of the first and last group balance structure based on RMSD.

The results indicate that the top three H-bond occupancies of apocynoside(I) interact with CYP2C9 and the top two among these amino acids are important (Figure 9(a)). The distance of each H-bond is consistent after an MD of 2 ns and these amino acids are at similar positions after superimposition (Figures 9(b) and 9(c)). Based on the above, we suggest that apocynoside(I) and CYP2C9 can become a stable complex and that apocynoside(I) can influence CYP2C9.

We note that 4-methoxymagndialdehyde is modified based on ligand RMSD and the top two H-bonds (over the threshold of occupancy); distances are presented in Figure 10. In these results, one of the important amino acids, Phe476, vigorously interacts with the ligand to create a balanced complex. This situation may indicate that the structure of 4-methoxymagndialdehyde can have H-bonds with adjacent amino acids; thus, the interaction will be extended.

The ligand RMSD indicates that the ligand has a variation of between 5 ns and 16 ns; otherwise, the H-bond distance is not transformed with the ligand (Figure 11). This condition confirms that the total energy and the complex RMSD do not have large amplitude after 5 ns. We suggest the main functional point of the ligand interacts with the protein but the benzene of the ligand cannot establish H-bond with the amino acids; thus, this benzene moiety will change position and angle to create a weak interaction. This result may indicate that prunasin, in the simulation site, will make the complex stable and prunasin will vary to induce a better complex.

**Figure 12:**
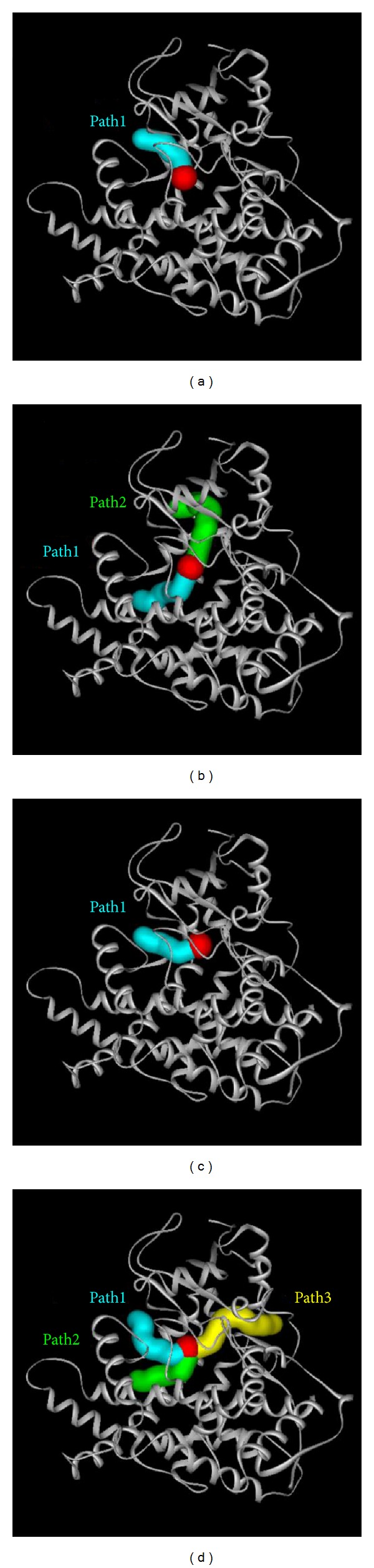
The pathway for ligand into the simulation site. The Cyan is number one path, the green is the second path, the yellow is the third path, and the red is ligand site: (a) warfarin, (b) apocynoside(I), (c) 4-methoxymagndialdehyde, and (d) prunasin.

The pathway of the ligand as it moves into the docking site is calculated based on the aperture of the protein and the molecular structure of the ligand (Figure 12). This result shows that prunasin has the largest pathway into the simulation site. We suggest that the structure of prunasin is not complicated and that the molecular weight, being less than others, induces the ligand to transform easily to give many pathways that can be traversed.

## 4. Conclusion

An important knowledge of personalized medicine and biomedicine, such as the analysis of regional disease [55], rare disease [56], clinical diagnosis cases [57, 58], and disease associated mutations [59–61] has been attracting more and more attention [62, 63]. The TCM is defined as a personal medicine. Our research applies a structure-based and ligand-based theory of CADD to screen TCM compounds for inhibition of CYP2C9. The selected compounds can take effect on CYP2C9 when the docking site does not consist of disordered regions. The efficacy of the selected TCM compounds is confirmed and this efficacy can indirectly prevent aging. After the analysis of MD and protein-ligand interactions, we suggest that apocynoside(I) and prunasin can make the complex balance faster but that 4-methoxymagndialdehyde has a more intense ligand dock in the protein. On the basis of the above observations, and compared to warfarin, we suggest that the selected compounds may have an effect on CYP2C9 inhibition.

## Supplementary Material

Table S1: The molecular structures of training set and test.
Table S2: The result of GA analysis for training set.

## Figures and Tables

**Table 1:** Scoring functions of the top compounds and the inhibitor of CYP2C9.

Compounds	Herbs	Predicted (pIC_50_)		Dock Score
		SVM	MLR	
Apocynoside(I)	*Apocynum venetum* L.	7.252	4.175	81.260
4′-methoxymagndialdehyde	*Magnolia officinalis* Rehd. et Wils.	6.211	7.681	60.282
Prunasin	*Citrus aurantium* L.	5.989	6.040	81.165
Warfarin*		5.957	13.085	40.014

*Control.
