# Supplementary material for: In Silico Investigation of Cytochrome P450 2C9 in relation to Aging Using Traditional Chinese Medicine
Source: Evid Based Complement Alternat Med. 2014 May 8;2014:404505. doi: 10.1155/2014/404505 (PMC4034651; doi:10.1155/2014/404505)
Supplement: Supplementary file 1 — Table S1: The molecular structures of training set and test. Table S2: The result of GA analysis for training set. [file 404505.f1.pdf]

Table S1. The molecular structures of training set and test set.

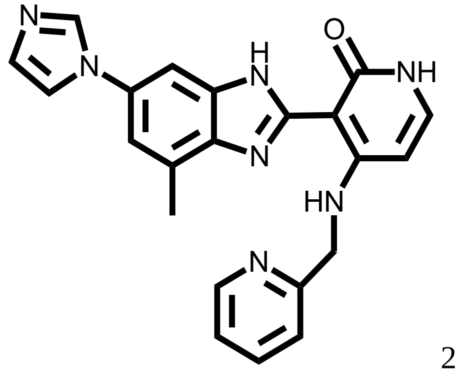

2

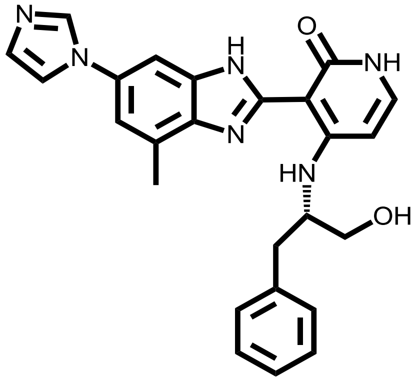

3

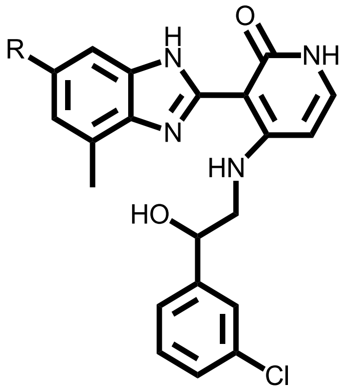

4,26-39

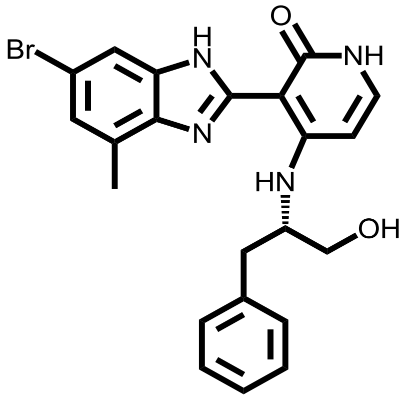

10

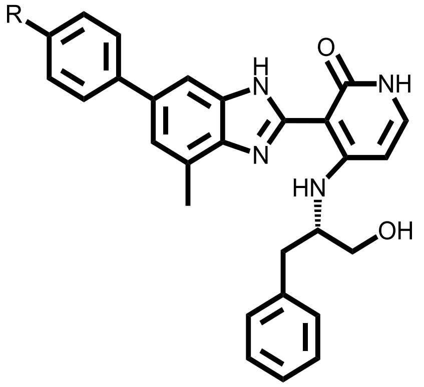

12-14

Name	R	IC_{50}(μM)
2	-	0.03
3	-	0.04
4	Imidazole	0.04
10	-	0.72
12	H	2.3
13	F	4
14	OMe	11
26	H	1.5

28	CONH2	2.9
29	CONHMe	2.8
30	CONH-cyclopropyl	8.1
31	CONH-cyclopentyl	3.5
32	CONH-cyclohexyl	6.6
34	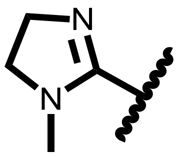	91
35	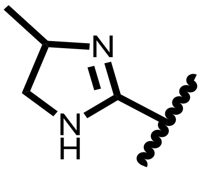	67
36	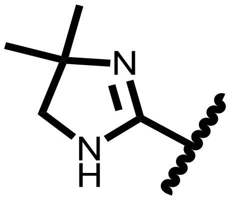	47
37	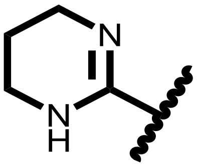	100
38	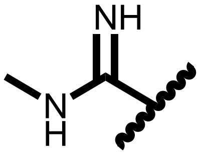	76
39	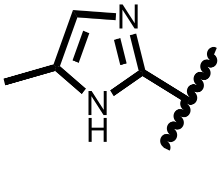	7.8

Table S2. The results of GA analysis

No.	equations	r^2
1	GFATempModel_1 = 9.3969 + 4.6637 * ALogP − 1.5025 * ES_Sum_dssC − 4.0939 * LogD + 0.0089932 * Jurs_DPSA_1 − 0.27175 * Jurs_WPSA_3	0.9581
2	GFATempModel_2 = 4.0101 + 3.5372 * ALogP − 1.1951 * ES_Sum_dssC − 3.3068 * LogD + 0.0049456 * Jurs_WNSA_2 + 6.5271 * Shadow_XYfrac	0.9339
3	GFATempModel_3 = 8.5756 + 4.3024 * ALogP − 1.3209 * ES_Sum_dssC − 3.8914 * LogD + 0.0036944 * Jurs_WNSA_2 − 0.078142 * Jurs_WPSA_3	0.9330
4	GFATempModel_4 = 7.8675 + 4.8378 * ALogP − 0.84583 * ES_Sum_dssC − 4.2679 * LogD − 0.19529 * Dipole_Y − 0.15792 * Jurs_WPSA_3	0.9319
5	GFATempModel_5 = 9.0886 + 4.6533 * ALogP − 1.4796 * ES_Sum_dssC − 4.2905 * LogD + 0.07713 * Jurs_WNSA_3 − 0.090269 * Jurs_WPSA_3	0.9257
6	GFATempModel_6 = 5.862 + 3.9645 * ALogP − 1.3775 * ES_Sum_dssC − 3.6419 * LogD + 0.0053017 * Jurs_WNSA_2 + 3.035 * Shadow_XZfrac	0.9239
7	GFATempModel_7 = 7.8349 + 3.5551 * ALogP − 1.5214 * ES_Sum_dssC − 3.3755 * LogD + 0.71979 * CHI_V_3_C + 0.0063963 * Jurs_WNSA_2	0.9234
8	GFATempModel_8 = 7.5854 + 3.6834 * ALogP + 0.040597 * ES_Sum_dO − 1.114 * ES_Sum_dssC − 3.4473 * LogD + 0.0065086 * Jurs_WNSA_2	0.9233
9	GFATempModel_9 = 190.57 + 5.1477 * ALogP − 1.1876 * ES_Sum_dssC − 4.3839 * LogD − 36.982 * CIC − 186.63 * SIC	0.9204
10	GFATempModel_10 = 8.1637 + 4.2035 * ALogP − 1.4532 * ES_Sum_dssC − 3.8642 * LogD − 0.70976 * CIC + 0.0053265 * Jurs_WNSA_2	0.9179
